# Characteristics of adolescents frequently restrained in acute psychiatric units in Norway: a nationwide study

**DOI:** 10.1186/s13034-016-0136-1

**Published:** 2017-01-12

**Authors:** Astrid Furre, Ragnhild Sørum Falk, Leiv Sandvik, Svein Friis, Maria Knutzen, Ketil Hanssen-Bauer

**Affiliations:** 1 Division of Mental Health and Addiction, Centre for Forensic Psychiatry, Oslo University Hospital, Oslo, Norway; 2 Centre for Child and Adolescent Mental Health, Eastern and Southern Norway, Oslo, Norway; 3 Oslo Centre for Epidemiology and Biostatistics, Oslo University Hospital, Oslo, Norway; 4 Division of Mental Health and Addiction, Department of Research and Development, Oslo University Hospital, Oslo, Norway; 5 Division of Mental Health Services, Akershus University Hospital, Lorenskog, Norway; 6 Division of Health Service Research and Psychiatry, University of Oslo, Oslo, Norway

**Keywords:** Adolescence, Health care research, Restraint, Inpatient

## Abstract

**Background:**

The use of restraints in adolescent psychiatric settings requires particular professional, ethical, and legal considerations. The purpose of this study was to investigate whether the number of restraint episodes per patient was related to any of several characteristics of the adolescents.

**Methods:**

In this nationwide study, we included all adolescents restrained during the period 2008–2010 (N = 267) in Norwegian adolescent acute psychiatric inpatient units. They constitute 6.5% of the adolescents hospitalized in these units in the same period of time. We collected data on the number of restraint episodes they experienced during the study period; Poisson regression was then used to analyze the impact of gender, social, mental health, and treatment characteristics on the frequency of restraint. We developed a risk index for the likelihood of experiencing multiple restraint episodes.

**Results:**

We found a skewed distribution of restraint episodes in which a small group (18%) of restrained adolescents experienced a majority (77%) of the restraint episodes. A large percentage of the restrained adolescents (36%) experienced only one restraint episode. Risk factors for multiple restraint episodes were female gender, lower psychosocial functioning (Children’s Global Assessment Scale below 35), more and longer admissions, and concomitant use of pharmacological restraint. Except for gender, we used these variables to develop a risk index that was moderately associated with multiple restraint episodes.

**Conclusions:**

As a small group of patients accounted for a large percentage of the restraint episodes, future research should further investigate the reasons for and consequences of multiple restraint episodes in patients at acute adolescent psychiatric units, and evaluate preventive approaches targeted to reduce their risk for experiencing restraint.

## Background

Various types of restraint are used in psychiatric institutions to stop patients from harming themselves, others, or property, including mechanical restraints, seclusion, pharmacological restraints, and physical holding. The use of restraint is potentially harmful, and thus the overall objective is to minimize its use. The use of restraint against adolescents requires particular professional, ethical, and legal considerations. The staff at many psychiatric units for adolescents consider the use of restraint to be unavoidable to manage severe aggressive behavior. In child and adolescent psychiatry, the use of restraint (especially physical holding) has sometimes been considered therapeutic, even if there is little evidence of such benefit [1]. Rather, studies have shown that patients often experience restraint as coercion and trauma, and that this results in less trustful relations with the staff [2]. Some patients have described flashbacks from prior traumatic events during physical holding [2–4], and some are physically injured [4, 5]. Use of restraint may also negatively influence the inpatient milieu [4].

A recent review of 49 studies of adult inpatient mental health services estimated that the prevalence of restraint was 3.8–20% and most frequently associated with male gender, younger age, foreign ethnicity, schizophrenia, involuntary admission, aggression or trying to abscond, and the presence of male staff [6]. Adult studies report substantial variability in the use of restraint between nations and hospitals [7–10]. Studies of restraint use in child and adolescent mental health services report relatively high rates, often at similar or higher levels compared to adult mental health services, and, again, the extent of the use varies considerably [11, 12]. One Finnish national study reported that about 40% of the adolescent inpatients had been restrained in some way during their admission [13]. Another Finnish study reported that 27% of the involuntary treatment periods in an 8-year period included the use of restraint and that there was considerable regional variation in the use of restraint [14]. In a previous paper based on the present study, we found that 267 (6.5%) of the 4099 adolescents admitted (voluntarily or involuntarily) to acute psychiatric units in Norway in 2008–2010 were restrained [15].

Another important finding in previous studies of restrained adolescents is the skewed distributions of restraint episodes, with small proportions of adolescents accounting for large proportions of episodes, and large proportions of adolescents being restrained only once or twice [16–21]. Two of these studies found that adolescents who had been restrained many times were younger [19, 20], and four studies found that adolescents who had been restrained more than once had longer hospital stays [17, 18, 20, 21]. One of these studies found that adolescents who had been restrained three or more times shared a particular profile: 67% had multiple admissions during the study period, all of them had a previous psychiatric hospitalization, and they were more likely to have lived in foster care, had special education, and a history of voicing suicidal ideation and attempting suicide [21]. Frequently restrained adolescents represent a specific challenge for the staff at inpatient psychiatric units, because the staff find the use of restraint necessary while acknowledging that there is the potential for physical and psychological harm with repeated episodes of restraint. Thus, for the sake of both the adolescents and the staff in such units, it is important to prevent the frequent restraint of adolescents. To our knowledge, no studies based on a nationwide sample have been published that identify the characteristics of adolescents who have experienced multiple restraint episodes in inpatient mental health services.

This paper presents data from a study on the use of restraint in acute psychiatric units for adolescents in Norway. We investigated whether the number of restraint episodes per patient was related to any of several characteristics of the adolescents. We also wanted to develop a risk index score based on our dataset to identify patients with higher risk for multiple restraint episodes.

## Methods

### Setting

We collected data from all of the Norwegian adolescent acute psychiatric inpatient units that are approved for involuntary admissions (N = 16), which included a total of 126 beds (mean 7.4, SD 2.9, range 2–14). These units provide inpatient mental health care mainly for adolescents aged 13–17 years, but they accept admission of younger adolescents if needed. Some adolescents are more than 17 years old at the time of discharge. During the study period, all of the units accepted around-the-clock emergency admissions. The units are distributed throughout Norway, and each unit has a uniquely defined catchment area. As a rule, drug-addicted adolescents are cared for by the child protection service. Three of the 16 acute psychiatric inpatient units were locked when needed, and the other 13 were permanently locked or had one permanently locked ward.

### Data collection

We collected data on all of the inpatients in the included units who experienced restraint from January 1, 2008 through December 31, 2010. We collected the data retrospectively during a nine-month period from August 2011 to May 2012. The first author visited all of the institutions and collected data on restraint episodes, demographic characteristics, and clinical variables. Information about restraint episodes was collected from routinely used handwritten restraint protocols. Other data were collected from the electronic patient records. The total number of admitted adolescent patients during the study period was retrieved from the electronic patient administrative system at each unit.

### Definitions of restraint in the Norwegian Mental Health Care Act

The Norwegian Mental Health Care Act regulates the practice of restraint procedures in Norway [22]. Staff members must consider less restrictive interventions first, and they cannot use restraint as a treatment. The following types of restraint may be used: (a) *mechanical restraints*, which inhibit the patient’s freedom of movement, including belts and straps and clothing specially designed to prevent injury; (b) *seclusion*, which refers to detention for a short period of time behind a locked or closed door without a staff member present; (c) *pharmacological restraint*, which refers to single doses of medicines that have a short-term effect and are used to calm or sedate a patient; and (d) *physical holding*, which refers to any technique in which staff members physically restrain a patient without using tools. Mechanical restraints and locked seclusion are not allowed for patients under the age of 16. Restraint can be used during either voluntary or compulsory admissions. All psychiatric institutions in Norway are obligated by law to have a restraint protocol in which each restraint episode is registered. The protocol describes the type and duration of the restraint and the reason for its use. Independent and authorized control commissions regularly checks all registrations in these protocols.

In this study, we did not include episodes of restraint that were needed for compulsory feeding in cases of severe anorexia (1896 restraint episodes distributed across 21 patients). The Norwegian Mental Health Act also regulates the use of compulsory feeding for patients with anorexia. These episodes are often included in the restraint protocols because wards may use mechanical restraints or physical holding to conduct forced feeding. However, whether or not these episodes are included in the protocols varies between wards.

### Data and variables

The dependent variable was the number of restraint episodes for each patient from all the admissions during the whole study period. The number of restraint episodes was categorized as 1, 2–4, 5–9, and ≥10. For adolescent patients with more than one admission in the three-year period, we collected data on the patient’s social and mental health characteristics from the most recent admission.

*Social characteristics.* We defined *immigrant background* as having two foreign-born parents, and coded this *Yes* or *No*. The variable *living arrangement* at the time of the most recent admission was coded in four categories: *living with both parents* (biological or adoptive), *living with one parent* (with or without stepparent), *living in foster care or institution*, and *other.* The variable *current involvement with the child protection service* was coded *Yes* or *No*.

*Mental health characteristics.* The local clinical teams assessed the adolescent patients’ conditions and coded their *main psychiatric disorder* using the Axis One (clinical psychiatric syndromes) in the multiaxial ICD-10 classification of child and adolescent psychiatric disorders from the World Health Organization [23]. Using this information, we grouped the adolescent patients into one of five categories based on their most recent admission (the ICD-10 codes are in parentheses): (1) *No Axis One disorder*; (2) *psychotic* (F20–29) *or pervasive developmental disorder* (F84); (3) *manic episode or bipolar affective disorder* (F30, F31.0–F31.2, F31.6–31.9); (4) *internalizing disorder* (depression F31.3, F32–33; anxiety F40–41, F93, F94; OCD F42; stress related F43; dissociative F44); (5) *externalizing disorders* (substance use F10, F12, F19; personality F60, F69; hyperkinetic F90; conduct F91–92; tics F95). *Global psychosocial functioning* was routinely rated by the clinicians at admission using the Children’s Global Assessment Scale (CGAS) [24]. We used the CGAS score from each patient’s most recent admission. The CGAS measures general functioning, with scores ranging from 1 (needs constant supervision) to 100 (superior functioning). We divided CGAS scores into three groups (tertiles): 1–34, 35–44, and 45–75. We did not measure the interrater reliability of the CGAS for this study. However, the interrater reliability of the CGAS in routine use was found to be moderate (intraclass correlation coefficient, .61) in a large study of clinicians in Norwegian outpatient child and adolescent mental health services [25].

*Treatment characteristics.* We divided the *number of admissions* in the study period into three groups (tertiles): 1, 2–3, and ≥4 admissions. We defined the *length of admission* as the number of days for the most recent admission and we divided this into three groups (tertiles): 1–4, 5–21, and ≥22 days. We coded *involuntary admission* as *Yes* if the patient was involuntarily admitted during the study period. We defined *concomitant use of restraint* as the use of pharmacological restraint in combination with any of the other types of restraint, and it was coded *Yes* when it occurred.

We developed a risk index score using the patient characteristic variables that were significantly associated with the number of restraint episodes (as indicated by the multivariate regression analysis). The categories for the variables were scored as 0, 1, or 2 (depending upon the number of possible response categories), with higher scores representing a stronger positive association with the number of restraint episodes. These scores were summed to make the risk index score. Because of the retrospective design of this study, and the fact that some of the variables required the completion of inpatient care, the prospective use of this risk index score at the patient level is limited. However, it may be useful to compare groups of adolescents admitted to inpatient care.

### Statistical analysis

Descriptive statistics are presented as frequencies and percentages. Zero-truncated Poisson regression analysis was applied to analyze the impact of gender, social, mental health, and treatment characteristics on the number of restraint episodes. We did not include *age* in our regression analyses; because each patient’s date of birth and exact age at the date of admission were unknown (only the age attained during the calendar year was available). In addition, adolescents must be at least 16 years old to be involuntarily admitted and to be restrained by mechanical means or seclusion. We performed univariate analyses for the independent variables: gender, immigrant background, living arrangement, current involvement with the child protection service, main psychiatric disorder, global psychosocial functioning (CGAS score), number of admissions in the study period, length of admission, involuntary admission, and concomitant use of restraint. Variables with *p* < .20 in the analysis were selected for inclusion in the multivariate model. Variables that were not statistically significant (*p* ≥ .05) in the multivariate analysis were deleted (largest *p* values first) until all of the remaining variables were significantly associated with the outcome. We used robust standard errors for the parameter estimates, as recommended by Cameron and Trivedi [26]. The effects are presented as incidence rate ratios (IRR) with 95% confidence intervals and p values. We tested the final model for multicollinearity by calculating the variance inflation factors for each of the independent variables. Estimated model fit is presented as pseudo R^2^ (explained variance). Because of the large number of missing values in CGAS (70 adolescents had no information), we reran the analyses omitting CGAS from the model to check for selection bias. We used the Goodman and Kruskal’s rank correlation statistics to measure the strength of association between the risk index score and the observed number of restraint episodes.

A p value <.05 was regarded as statistically significant. All statistical analyses were conducted using SPSS (version 18.0) and Stata [27, 28].

## Results

### Study sample

The sample comprised 267 adolescents who had experienced one or more restraint episodes during the 3-year study period (2008–2010). This group of 267 adolescents constitutes 6.5% of the 4099 adolescents admitted one or more times to the units in the same period. The mean age of the sample was 16.0 years (SD 1.8, range 10–21), 158 (59%) of whom were female. Seven of the adolescents were 10 or 11 years old, and eight were 19–21 years old. Eighteen (7%) of the adolescents experienced concomitant pharmacological restraint, most of them during episodes of physical holding. One adolescent was pharmacologically restrained in combination with seclusion and three in combination with mechanical restraint. The sample is described in detail in Table 1.

**Table 1:** Relationship between patient characteristics and the number of restraint episodes

	Number of restraint episodes				N
	1	2–4	5–9	≥10	
	N (%)	N (%)	N (%)	N (%)	
Total sample (patients)	97 (36)	88 (33)	35 (13)	47 (18)	267
Gender					
Female	59 (37)	49 (31)	17 (11)	33 (21)	158
Male	38 (35)	39 (36)	18 (17)	14 (13)	109
Social characteristics					
Immigrant background					
Yes	11 (31)	15 (43)	4 (11)	5 (14)	35
No	79 (37)	69 (32)	27 (13)	38 (18)	213
Missing	7 (37)	4 (21)	4 (21)	4 (21)	19
Living arrangement					
With both parents	32 (37)	26 (30)	8 (9)	21 (24)	87
With one parent (with or without stepparent)	26 (41)	23 (37)	9 (14)	5 (8)	63
Foster care or institution	36 (34)	36 (34)	16 (15)	19 (18)	107
Other	1 (14)	3 (43)	1 (14)	2 (29)	7
Missing	2 (67)	0 (0)	1 (33)	0 (0)	3
Current involvement with the child protection service					
Yes	40 (33)	43 (35)	19 (15)	21 (17)	123
No	37 (35)	33 (31)	12 (11)	23 (22)	105
Missing	20 (51)	12 (31)	4 (10)	3 (8)	39
Mental health characteristics					
Main psychiatric disorder (Axis One only^a^)					
No Axis One disorder	23 (58)	9 (22)	5 (13)	3 (8)	40
Psychotic or pervasive developmental disorder	19 (50)	9 (24)	2 (5)	8 (21)	38
Manic episode or bipolar affective disorder	4 (21)	7 (37)	5 (26)	3 (16)	19
Internalizing disorder	28 (29)	33 (35)	15 (15)	19 (20)	95
Externalizing disorder	23 (31)	30 (40)	8 (11)	14 (19)	75
CGAS^b^					
1–34	15 (23)	24 (37)	8 (12)	18 (28)	65
35–44	23 (37)	22 (35)	8 (13)	9 (15)	62
45–75	30 (43)	19 (27)	8 (11)	13 (19)	70
Missing	29 (41)	23 (33)	11 (16)	7 (10)	70
Treatment characteristics					
Number of admissions in the study period					
1	46 (46)	39 (39)	8 (8)	8 (8)	101
2–3	27 (31)	30 (34)	15 (17)	15 (17)	87
4–19	24 (30)	19 (24)	12 (15)	24 (30)	79
Length of admission (days)					
0–4	41 (52)	23 (29)	8 (10)	7 (9)	79
5–21	31 (30)	38 (37)	18 (17)	17 (16)	104
22–425	25 (30)	27 (32)	9 (11)	23 (27)	84
Involuntary referral					
Yes	28 (27)	30 (29)	17 (16)	29 (28)	104
No	66 (42)	54 (35)	18 (12)	18 (12)	156
Missing	3 (43)	4 (57)	0 (0)	0 (0)	7
Concomitant use of restraint					
Yes	3 (17)	5 (28)	2 (11)	8 (44)	18
No	94 (38)	83 (33)	33 (13)	39 (16)	249

^a^Axis One is the clinical psychiatric syndromes [23]

^b^ *CGAS* Children’s Global Assessment Scale (global psychosocial functioning)

### Distribution of restraint episodes

**Fig. 1:**
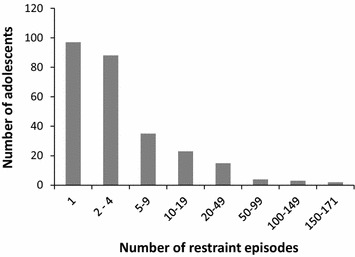
Distribution of 2277 restraint episodes among 267 adolescents

The adolescents in our sample experienced 2277 restraint episodes (78.7% of these were psychical holding, 13.4% mechanical restraint, 5.9% seclusion, and 1.6% pharmacological restraint) during the study period. The median number of restraint episodes per patient was two (range 1–171). Figure 1 shows that 97 (36%) of the adolescents were only restrained once, 88 (33%) 2–4 times, 35 (13%) 5–9 times, and 47 (18%) 10 times or more. This latter group accounted for 1762 (77%) of the restraint episodes.

### Regression analysis

The results from the regression analysis are presented in Table 2. The univariate analyses showed that having an Axis One disorder, lower global psychosocial functioning (CGAS below 35), more admissions in study period, an admission of 22 days or longer, and concomitant use of restraint were associated with more restraint episodes, whereas living with one parent was associated with fewer restraint episodes than living with both parents. The other variables were not significantly associated with the number of restraint episodes.

**Table 2:** Risk factors for more frequent restraint episodes

	Unadjusted model			Adjusted model		
	IRR^b^	95% CI^a^	*p*	IRR^b^	95% CI^a^	*p*
Gender (ref: male)						
Female	1.69	.99–2.90	.055	2.04	1.1–3.74	.021
*Social characteristics*						
Immigrant background (ref: yes)						
No	1.09	.37–3.18	.876			
Living arrangement (ref: with both parents)						
With one parent (with or without stepparent)	.42	.19–.94	.034			
Foster care or institution	.81	.42–1.57	.530			
Other	1.18	.40–3.46	.767			
Current involvement with the child protection service (ref: no)						
Yes	.85	.46–1.57	.610			
*Mental health characteristics*						
Main psychiatric disorder (ref: no Axis One disorder)						
Psychotic or pervasive developmental disorder	4.08	1.50–11.09	.006			
Manic episode or bipolar affective disorder	6.45	1.93–21.52	.002			
Internalizing disorder	3.85	2.09–21.52	<.001			
Externalizing disorders	3.74	1.99–7.05	<.001			
Global psychosocial functioning						
CGAS (ref 45–75)						
1–34	2.35	1.27–4.35	.006	2.59	1.34–5.04	.005
35–44	1.48	.67–3.26	.334	1.70	.71–4.11	.236
*Treatment characteristics*						
Number of admissions in the study period (ref: 1)						
2–3	2.33	1.07–5.09	.034	2.32	1.08–4.95	.030
4–19	2.78	1.40–5.51	.003	3.28	1.76–6.13	<.001
Length of last admission in days (ref: 1–4)						
5–21	1.12	.55–2.28	.757	1.02	.51–2.03	.956
22–425	2.43	1.14–5.20	.022	2.63	1.21–5.66	.014
Involuntary referral (ref: no)						
Yes	1.70	.93–3.08	.083			
Concomitant use of restraint (ref: no)						
Yes	3.97	2.01–7.84	<.001	3.44	1.90–6.22	<.001

Based on zero-truncated Poisson regression analysis

^a^ *CI* confidence interval

^b^ *IRR* incidence rate ratio

The multivariate analysis indicated that being female, lower global psychosocial functioning (CGAS below 35), more admissions in the study period, an admission of 22 days or longer, and concomitant use of restraint were associated with more restraint episodes. We found no evidence of multicollinearity in the multivariate regression analysis. Omitting CGAS from the model gave similar results (data not shown). The R^2^ of the adjusted model was 31.9%.

### Risk index score

The five significant variables from the multivariate model were considered for inclusion in the risk index score (risk for more restraint episodes). However, we did not include gender in the risk index score, because the finding that females have a greater risk of restraint episodes is not generally supported in the literature, and this variable had the lowest IRR. The remaining four variables were included in the risk index score, and the scoring system is shown in Table 3. The categories for the risk index score were tabulated based on the number of restraint episodes per patient (Table 4). The likelihood of experiencing 10 or more restraint episodes increased from 0% when the risk index score was ≤1 to 35% when the risk index score was ≥4. The Goodman and Kruskal’s correlation was .35.

**Table 3:** Scoring system for the risk index score

Variable	Results	Scores
Global psychosocial functioning (CGAS)	1–34	2
	35–44	1
	≥45	0
Number of admissions	1	0
	2–3	1
	≥4	2
Length of admission (days)	1–4	0
	5–21	1
	≥22	2
Concomitant use of restraint	No	0
	Yes	1

Possible range of the risk index score: 0–7

**Table 4:** Relationship between risk index score and restraint episodes

Risk index score	N (%)	Number of restraint episodes			
		0–1	2–4	5–9	≥10
		n (%)	n (%)	n (%)	n (%)
0–1	28 (100)	15 (54)	10 (36)	3 (11)	0 (0)
2	38 (100)	16 (42)	11 (29)	5 (13)	6 (16)
3	63 (100)	23 (37)	22 (35)	8 (13)	10 (16)
4–7	68 (100)	14 (21)	22 (32)	8 (12)	24 (35)

Goodman and Kruskal’s gamma is .35

Data from N = 197 adolescents with valid Information for all four of the variables used for the risk index score

## Discussion

We found a skewed distribution of restraint episodes. A small group of restrained patients (18%) who were restrained more than 10 times accounted for the majority (77%) of the restraint episodes. The multivariate analysis revealed that the adolescents who were likely to experience more restraint episodes were female, with lower global psychosocial functioning, more and longer admissions, and those who had experienced concomitant use of restraint. Four of these five variables (excluding gender) were used to construct a risk index score, with higher scores indicating a greater risk of restraint episodes.

Earlier studies have also found highly skewed frequencies of restraint. One US study found that seven (1.7%) out of 408 adolescents experienced 56.6% of 1349 aggressive episodes requiring an intervention [17]. A study on an adolescent inpatient unit reported that 7.4% of the secluded patients experienced 81% of the seclusion episodes, and two of these patients (1.3%) experienced 45% of the seclusion episodes [16]. Another large retrospective study collected data on all restraint episodes during 7.5 years in a US psychiatric hospital for children, adolescents, and adults with severe mental illness. They found that 20, 10, and 1% of the most-often restrained patients experienced 75, 61, and 21% of the restraint episodes, respectively [29].

### Gender

We found that females had a greater risk of multiple restraint episodes. A study of adolescents in two US residential treatment centers found that adolescents with moderate and high levels of seclusion and restraint were more often black and/or female [30, 31]. However, there are studies of adolescents with different findings that give a more inconclusive picture of the evidence [17, 18, 21, 32]. A recent adult study from Norway found slightly more females than males among the frequently restrained patients, but the difference was not statistically significant [33]. A reason for the inconclusive picture may be that different nations treat different adolescent populations in their psychiatric inpatient units. There may also be different pathways to multiple restraint episodes for female and male adolescents which are not controlled for in the different studies.

### Social characteristics

None of the social characteristics (immigrant background, living arrangement or current involvement with the child protection service) increased the risk of multiple restraint episodes in the multivariate model. Immigrant background was a significant predictor of being restrained compared to not being restrained in our previous paper, while the other two social characteristics were not [34]. We have not found other studies that analyzed these characteristics as risk factors for multiple restraint episodes.

### Mental health characteristics

We found that that lower global psychosocial functioning (CGAS below 35) was associated with more restraint episodes. One study has reported that adolescent inpatients with more than 50 aggressive episodes requiring intervention had a lower mean global psychosocial functioning at admission [17].

Having an Axis One psychiatric disorder was not a significant risk factor in the multivariate analyses. A previous study of female adolescents found that externalizing or internalizing disorders (versus no diagnosis) predicted restraint episodes, even in a multivariate regression analysis [35]. Another study did not include diagnoses in the multivariate analyses because of high multicollinearity with admission status (court ordered, physician’s emergency certificate, or voluntary) [36].

### Treatment characteristics

Our finding that more and longer admissions are associated with a greater number of restraint episodes has been reported in three previous US studies [17, 21, 35].

In the present study, involuntary admission was not a risk factor for multiple restraint episodes. One US study found that admission status was the strongest and most consistent predictor of frequent restraint [36]. However, their admission procedures (court ordered, physician’s emergency certificate, or voluntary) differ from those in Norway.

In our study, 7% of the restrained adolescents experienced concomitant use of restraint. A recent Finnish study found that 8.5% of the mechanically restrained adolescents received intramuscular medication during the restraint episode [37]. However, they did not analyze whether this concomitant use was a predictor for more restraint episodes. In the present study, we found that the use of concomitant restraint was a strong risk factor for frequent restraint episodes. As far as we know, this has not been reported previously. One rationale for using concomitant restraint is that it shortens the duration of restraint [17], but we have not found any studies that document this effect. Our finding indicates that this group deserves special attention to prevent the use of restraint in clinical practice.

### Risk index score

The risk index score that we developed was moderately associated with a larger number of restraint episodes. The risk index score is easy to calculate and it may be useful in clinical practice. However, these results should be replicated in other samples before the risk index score can be implemented in clinical use.

### Strengths and limitations

A methodological strength of our study relative to previous studies is the highly representative national sample. We used a 3-year cohort of all of the adolescent inpatients who had experienced restraint in all of the acute psychiatric units in Norway. In addition, we had access to data for all of the registered restraint episodes they experienced during that period.

One limitation of this study is the retrospective design, which leaves us unable to determine the reliability of the clinical data, such as the main psychiatric disorders, the CGAS scores, and the reasons for the use of restraint. A second limitation is the lack of a reliability test of the data extraction. We are not aware whether our findings had been different if we had collected data on social and mental health characteristics from another admission than the most recent, i.e. the first.

## Conclusion

Based on a large and representative national dataset, a notable finding in this study is the skewed distribution of restraint episodes. A small group of frequently restrained adolescent patients (18%) accounted for a large percentage (77%) of the restraint episodes. Conversely, a large percentage of the restrained adolescents (36%) were only restrained once. Factors associated with more restraint episodes were female gender, low global psychosocial functioning, longer and more admissions, and concomitant use of pharmacological restraint. A risk index score based on four variables was moderately associated with more restraint episodes in our sample. If these findings are replicated in other samples, the identification of patients at high risk of multiple restraint episodes may inform the development of interventions to reduce the use of restraint. The effects of such interventions should be evaluated in well-designed future research trials.

## Abbreviation

CGAS: Children’s Global Assessment Scale
